# Inflammation of the Periosteum, &c. Fatal, with Four Cases

**Published:** 1831-04-01

**Authors:** 


### CLINICAL REVIEW,

OR,

### HOSPITAL REPORTER;

WITH

### CRITICAL COMMENTARIES.

### I.

### GLASGOW ROYAL INFIRMARY.

#### Inflammation of the Periosteum and deep-seated Cellular Membrane, ending in Death of the Bone or Destruction of Joints.

We have always characterised the surgical reports from the Glasgow Royal Infirmary as very useful and practical papers. Dr. Weir has followed the example of his predecessors and contemporaries, and published some valuable facts and instructive cases, in the Number of our Glasgow contemporary for November of the present year. We select, on this occasion, the subject of periostitis, as Dr. Weir denominates it. We know that the affection of which we are about to detail instances, is not uncommon, we have seen several such ourselves, and we know those who have seen others. Yet it is not an affection of which we have a good description in any surgical works which we have seen.

*Case* 1. John Henderson, æt. 10, admitted Dec. 4th, 1829.

Left knee much swollen, measuring four inches more than the opposite—swelling soft and puffy—joint exceedingly painful on motion or when touched. Immediately over the external condyle, an ulcerated opening, discharging much purulent matter not communicating with the joint, but allowing a probe to be passed for several inches upwards along the femur, which is felt bare. Whole thigh much swollen to the groin—most severe pain in the knee-joint and lower half of the thigh on the slightest motion of the limb, but little pain when at rest. Acute pain along the front of the tibia, with œdema of the leg and foot—on the dorsum of the foot a slough, which is extending. General health much impaired—pulse 120, feeble—great emaciation. Six weeks ago he received a blow upon the knee, immediately followed by pain and swelling of the joint and lower part of the thigh. Two weeks ago, an abscess formed over the external condyle, which was punctured, and eight ounces of bloody matter were discharged. Wine, tonics, roller and compresses were employed, an incision was made on the front of the tibia and dorsum of the foot. The discharge in-increased greatly, and was very fetid, the slough separated from the foot, the debility became extreme, and three weeks after admission he died. Several incisions about the knee-joint were required; the discharge throughout was thin, dark, offensive; and before death the whole cellular membrane of the leg was in a state of slough.

*Dissection.* Much sero-purulent fluid in the cavity of the pleura, with slight traces of inflammation of that membrane, and apparently recent adhesions. Substance of the lungs healthy, excepting an abscess containing sero-purulent matter, in a small portion of the left lung. Much serous fluid in the abdomen, and mesenteric glands enlarged. Left femur diseased throughout; the lower half in a state of necrosis, with a scale of bone of some thickness beginning to separate—the disease extending for some way into the medulla, and in several places near the condyles. New osseous particles deposited in irregular patches on its surface; on the upper half of the femur many pieces of new bone deposited, forming irregular prominences in some parts, but chiefly smooth and uniform, looking like a layer of new bone, and augmenting the circumference of the femur. Cartilages of the knee-joint completely destroyed, and the ends of the tibia and fibula softened. Shaft of the tibia nearly healthy.

"I believe this case to have been originally one of acute inflammation of the periosteum of the femur, followed by the formation of matter, both above and below that membrane, and attended by a very different affection, viz. ulceration of the cartilages of the knee-joint. It is likely that the blow upon the knee, besides injuring the shaft of the femur, had immediately induced inflammation of the joint, followed by ulceration and destruction of the cartilages of the tibia and fibula, with caries of the heads of these bones. It will be remarked that the disease of the femur was strictly confined to the shaft, the spongy heads of the bone remaining healthy; and it had gone so far that a commencing separation, between the cylinder and the epiphysis of the bone, at the knee-joint, was visible. With respect to the upper part of the femur, it does not appear to me that this portion was wholly necrosed and a new bone formed, completely surrounding or incasing the old, but that merely a thickening or enlargement of the old took place; a hyperostosis, as it has been called."

*Case* 2. George Stirling, æt. 8, admitted Jan. 2, 1830.

Eleven days previously, after the general symptoms of inflammatory fever, he was seized with severe pain and slight swelling of the left leg, immediately over the front of the tibia, in fact, with well-marked symptoms of inflammation of the periosteum. There was no discoloration of the integuments, no tension, little swelling, but deep-seated, excruciating pain, aggravated on the slightest motion or pressure, and high fever.

"An incision was made on front of tibia, three inches in length, down to the bone, which gave exit to a considerable quantity of blood, but no matter. The limb was ordered to be kept constantly cool with evaporating lotion. Next day there was some redness of the leg, but the pain was not so severe. An abscess had formed above the knee, which was opened, and several ounces of pus let out. There was now some pain of opposite leg and knee-joint, to which leeches were applied. The fever was less, his bowels had been opened, and he had got some sleep. On the 4th Jan. the third day after admission, it was evident that suppuration had taken place in both legs; the former incision on left was therefore enlarged, and a small incision made on right, near the head of the tibia, and healthy matter discharged from both. The discharge from the thigh was profuse, thin and unhealthy; the suppuration involved its lower half, the probe passing for several inches upwards, close to the femur, which was felt rough. Both tibiæ, at the places incised, were also exposed, denuded of periosteum. Wine, quinia, and nourishing food, were now liberally given. The left leg, where the disease was most extensive, presented the appearance of an œdematous limb, studded over here and there with red patches. Several incisions were made for the discharge of matter, and an opening was also made on the dorsum of foot, where sloughing soon took place, exactly as occurred in the boy Henderson. In the right leg the inflammation was completely arrested by the first incision; the tibia was bare for about an inch and a half only, and to this portion the disease seemed to be confined. The general health soon gave way under so much local irritation and discharge; the disease extended, and, before he died, it showed itself at the very top of the left femur, where an opening required to be made over great trochanter, which discharged a large quantity of highly fetid pus. After this he sunk rapidly, and died on 25th January, twenty-four days from the appearance of periostitis."

*Dissection.* Necrosis of inferior third of left femur, and upper third of left tibia; affection distinctly confined to the shaft, the spongy heads of the bones, and the joints remaining healthy. The disease seemed to involve the whole cylinder of the bones, and there was an evident commencing separation of the shaft from the extremities. At the upper part, where the abscess had been opened, the bone was deprived of periosteum and a small portion of it dead. Upper third of right tibia in a state of necrosis, the head remaining healthy. No attempt any where to form new bone. Abdominal and thoracic viscera sound.

"This case was also a very well-marked instance of acute inflammation of the periosteum, proceeding rapidly, and terminating in the death of the bone. It was unusual, from the circumstance of the disease affecting the tibia and femur of the one leg, and the tibia of the other, all at the same time; thus indicating some affection of the system, sufficient to entitle us to say that it was a constitutional disease. Although the tibia was the part first complained of, it will be remarked that suppuration took place first in the thigh. It is probable that the disease commenced in both situations simultaneously. Early incision was here evidently of great advantage in arresting the disease in the right leg, in which its progress was so different from that of the left. The appearance of the latter was very similar to that of Henderson, but the case differed from his in there being no affection of the joint, no caries of the spongy extremities of the bones, and no thoracic or abdominal disease. It was different also in there being no attempt to form new bone. It would appear that there was not sufficient time for this effect to be produced, the disease having continued only for three weeks. In Henderson it existed for nine weeks."

*Case* 3. Philip Kelly, æt. 12, admitted Nov. 19, 1829.

Left thigh measuring four inches in circumference more than right—inferior half of swelling hard, irregular, acutely painful on pressure, and appearing to arise chiefly from bone. On inner side of thigh, about two inches above knee-joint, an opening through which the probe can be passed for nine inches upwards, and the femur distinctly felt bare to some extent; this opening, with another lower down, discharging fetid purulent matter in abundance. Thigh not painful on rest, very much so on motion. No affection of knee-joint. Has had disease about the femur attended with slight discharge of matter for six years, but it gave him very little uneasiness till three weeks ago; the discharge then stopped, he was immediately seized with rigors followed by fever, and an acute abscess formed on the thigh, which was opened the day before his admission and a pint of healthy pus discharged. The opening was now enlarged, the limb bandaged from above downwards, the matter carefully pressed out daily, compresses applied, and wine and nourishing diet allowed. Under this treatment matters went on well till the 29th, ten days after admission, when he was seized with severe pain on the front of the tibia a few inches below the knee. The fever grew higher, but repeated bathing, with cold applications, removed the affection in a few days. The disease in the thigh improved, and at the end of five weeks, the boy was discharged with the discharge very trifling, the opening nearly closed, and the bone not felt by the probe. The bony enlargement continued, but the lad could walk. On the 27th January, four weeks after his dismissal, he returned with the limb very nearly in the same state as on his former admission. The opening had closed up, the matter accumulated, acute inflammation supervened, and a new incision was required. The discharge was copious and healthy, the probe could be passed upwards for four inches and a half, and the bone was felt bare. There was some redness and swelling of the integuments around the opening, the pain on pressure was severe, the pulse 120, and the skin hot. The same treatment was adopted, the swelling and pain gradually abated, and the discharge became less. Several small scales of bone were thrown off, and another opening took place on the opposite side of the thigh. On the 15th Feb. he was again dismissed, relieved, the discharge being very scanty and the general health good. We think that the following remarks by Dr. Weir will not be unacceptable to our readers. They appear to us to be, generally, very correct.

"At the time this case was under treatment, I had an opportunity of seeing another in private, in which the disease proceeded almost exactly in the same manner; and a few weeks ago, I had under my care, for some time, a boy also with the same symptoms. The history and progress of these three cases were very nearly the same, in regard to the age and constitution of the patients, and the seat and termination of the disease. Two of them were similar to each other, also, in the fact that there appeared a disposition to the disease, in more than one bone at the same time.

It is unnecessary to detail the other two cases which occurred during the winter. They were instances of the disease affecting the leg, in the one the tibia, in the other the fibula, and proceeding in the way it most generally does, viz. to superficial necrosis and exfoliation of the bone. The first presented the general appearances of what is often called erysipelas, but the symptoms which are characteristic of that disease, and which were present in that case on admission, viz. swelling, redness, tension and pain of the part, I am satisfied are not present in periostitis, until the disease has reached the stage of suppuration; whereas, in the true erysipelas, such are the most prominent symptoms, not only before any matter has formed, but also in many cases where suppuration does not take place at all. The one was a girl aged 14, the other a lad aged 17. The disease in both went on rapidly to the formation of matter. It was, however, kept within moderate bounds, by early and free incisions, without which there is no doubt the inflammation and suppuration, at least of the soft parts, would have extended greatly. In both, several scales of bone were thrown off before they left the hospital, and when dismissed there remained a considerable enlargement of the bone, and a small sinous ulcer of the integuments, but the disease gave little or no trouble.

It may be remarked, that in all these cases the patients were young, four of them being between 8 and 14 years of age, and one 17. The whole were scrofulous subjects, the two first very much so; and in several of them, the disease assumed so much of a constitutional tendency, that more bones than one were affected almost at the same time. This was the case with Stirling, in whom one femur and both tibiæ were affected; in Kelly and Henderson, in whom the femur and one tibia were affected; and in the girl, there was a tendency to suppuration in more than one place, she having an abscess on the chest, and another on the arm, besides the affection of the leg. I may likewise mention that in one of the cases alluded to, but not detailed, there was an abscess formed at the lower end of the tibia, while periostitis existed in the femur. It may also be remarked, that in several of these cases the disease was the result of external injury,—that when the femur was the part affected, it uniformly commenced at its lower part, and spread upwards, and that it was very generally confined to the shaft of the bone, the head remaining healthy. In the two cases which proved fatal, but more particularly in case 2d, there appeared a distinct attempt by nature to separate the shaft from the epiphysis of the bone, thus most effectually limiting the disease.

I have named the disease in these cases Periostitis, because I consider that in all of them the affection commenced in the periosteum, although it is probable that the disease was not altogether confined to that membrane, but extended, at least in some of them, to the bone itself. If, however, the bone was affected at all, in the first instance, the inflammation was confined to its surface, as in none of the cases was there produced necrosis or death of the whole cylinder of the bone, where a new shaft is formed and completely surrounds the old, from one epiphysis to the other. The only case where the disease extended any way into the medulla of the bone was the first, where, certainly, on a cursory view of it, the upper third of the femur had very much the appearance, as if a perfect circle of new bone had been formed, and surrounded the old. This, however, on more accurate examination, I am satisfied, was not the case.

In inflammation of the periosteum that membrane becomes thicker, more vascular, and loses its transparency. It is probable that in the most acute cases both the upper and under surfaces are affected, and the pus, being almost immediately formed, makes its way outward, and affects the soft parts much sooner than occurs when the matter is situated only beneath the membrane. Sir A. Cooper says, that when this takes place, it is a long time before the periosteum ulcerates; and the disease has not the characters of an acute affection.* It would appear, that in acute periostitis effusion of sero-purulent matter takes place very early, and the membrane is detached from the bone to a greater or less extent, according to the extent of the inflammation. Suppuration, however, does not necessarily take place in mild cases; and when it does, and the periosteum, is separated, the bone does not necessarily die. In the boy Kelly, although very well-marked symptoms of acute periostitis of the tibia occurred, these, by leeching and cold applications, were subdued, and no suppuration followed; and I have seen two cases of the disease in the femur ending in profuse suppuration, without any exfoliation of bone. When the symptoms are well-marked, and no suppuration takes place, I would consider that the inflammation had been confined to the upper surface of the periosteum.

The pain in periostitis is deep-seated, very severe, felt along the whole shaft of the bone, and subject to exacerbations. Sometimes when it affects the tibia, or any bone with slight covering of the soft parts, there is a blush of redness on the skin, and very soon, effusion into the cellular substance. In general, however, particularly in the thigh, it is some time before the integuments show any appearance of disease; and if no incisions are made, it is long after suppuration takes place, before the matter reaches the surface. The limb assumes more of an œdematous appearance, and the whole cellular substance is infiltrated before the integuments give way; hence the necessity for early incisions. The skin at last inflames, and ulcerates in various places. If the patient survive the effects of the profuse suppuration and great destruction of parts, and the disease lapse into a chronic stage, spongy granulations spring up at these various openings, and they usually become fistulous sores leading to the diseased bone.

It is worthy of remark, the different appearances which a limb presents on the first invasion of periostitis, from that exhibited when the inflammation is in the skin, or in the fascia. In the former there is no redness of the integuments; there is very little swelling; there is no tension whatever; but there is excruciating deep-seated pain. This last, therefore, is the most prominent symptom in this affection. The absence of redness of the integuments, while there is such acute pain, has got for it, among the common people, the name of the 'White Rose.' I speak of the disease at its *commencement*. In inflammation of the parts not so deep-seated, as of the fascia, or of the parts immediately below the fascia, there will be very great tension; there will be more swelling and some superficial redness, and probably serous effusion; but the pain, though great from the tension and binding down of the fascia, will not be so acute as in the former case. Here the most prominent symptom is the tension or firm swelling of the part. While again, in affections of the parts still more superficial, as of the skin, or of the tissue immediately below it, the principal diagnostic mark is the great redness of the integuments, —the fiery redness, the burning or itching pain, and the loose swelling, without so much tension as in the last case. In this third species of inflammatory affection, the redness is the most distinctive feature. Now all these inflammations have been named by different authors erysipelas, particularly the two last; because these are often combined

* "Cooper's Lectures, p. 630."

by the time the surgeon is called in. Hence the great confusion and difference of opinion with regard to the seat, appearances, and treatment of erysipelas. I think it is Mr. Copland Hutchison who says, that erysipelas sometimes commences in the periosteum. The term erysipelas should be confined to the last-described affection, dividing it of course into the simple and phlegmonous, according as it is quite superficial, or more deeply situated; the second mentioned should be called fascial or subfascial inflammation; and when the periosteum is affected, periostitis is a very proper appellation.

The cases given above exhibit the disease in its *acute* form. When the disease is of a more chronic character, it commences more insidiously, proceeds very slowly, and I think does not so generally terminate in suppuration, or in destruction of the bone. It is more frequently succeeded by a very considerable thickening of the membrane, or an enlargement of the bone itself, which may remain stationary and without much disturbance of the system for many years, unless it be brought into a state of acute inflammation, in consequence of external injury. Such cases, besides, are not attended with fever or much constitutional irritation. They not unfrequently depend on a syphilitic taint, but are often enough met with unconnected with that disorder. The following appears to have been a case of this kind."

*Case* 4. Elizabeth M'Lean, æt. 21, admitted March 10th.

Whole anterior aspect of middle third of left tibia occupied by a diffused, very firm, indurated swelling, intimately connected with the bone, and acutely painful on pressure, the pain shooting up towards the knee, and most severe during the night; integuments not discoloured; general health unaffected. Five weeks ago she fell and struck the leg against a stone. Little pain was felt at the time, but in a few days afterwards pain and swelling commenced, and have since been increasing. The treatment consisted of leeches, alterative aperients, and blisters. She was dismissed, but returned in a few days complaining of the same deep-seated pain particularly severe during the night, and preventing rest. The upper third of the tibia was now considerably enlarged and acutely painful on pressure. Camphorated mercurial ointment was rubbed on the part morning and evening till the skin became tender, when calomel and opium were given. This was alternated with leeching and blistering, but the disease increased slowly, and an incision, three inches long, was made about the middle of the tibia down to the periosteum, with relief to the pain. No pus was discharged, but a considerable quantity was obtained from another incision in the upper third of the leg. The mouth was slightly affected by mercury. Poultices were applied, the pain in the leg was removed, the wounds granulated and contracted, and were cicatrized on the 8th June, with the exception of a small fistulous opening, through which the probe passed, apparently, into the shaft of the tibia. This was enlarged, but no bone came away whilst she remained in the house. On the 2d July she was dismissed, the health being pretty good, the pain in the leg entirely gone, but a copious discharge continuing from the wound over the tibia. In September the sinus was perfectly closed; no bone had come away, but the whole tibia remained somewhat thickened; the health was good.

Inflammation of the periosteum and deep-seated cellular membrane is far from uncommon. We have seen it many times in the thigh, more rarely in the leg, occasionally in the upper extremities. In the thigh it occasionally puts on a very severe form. The lower part of the thigh and the knee-joint swell more or less suddenly, acute pain follows, then there is effusion into the joint and into the cellular membrane of the limb, suppuration follows within and without the articulation, the cartilages ulcerate, the periosteum is separated from the femur, and the patient dies worn out with pain, and hectic. Of this we have witnessed several examples, and in most, the affection appeared to be rheumatic, or at least to be produced by cold. We could mention some interesting facts, but our limits forbid us to enter on a subject that would require more space than we can afford. One point we cannot forbear adverting to, and that is the employment of mercury in these affections. Calomel and opium are most important remedies in the acute inflammation of the periosteum and deep cellular membrane. The only recoveries, so far as we recollect at this moment, which we have witnessed, have been in cases in which it was employed. We are speaking of the affection of the thigh. Of course it must be combined with numerous leeches, and so forth. We are rather surprised that Dr. Weir makes little or no mention of the effects of mercury in these cases.

---

